# Is serum hemoglobin level an independent prognostic factor for IgA nephropathy?: a systematic review and meta-analysis of observational cohort studies

**DOI:** 10.1080/0886022X.2023.2171885

**Published:** 2023-01-30

**Authors:** Kang Zhang, Meng-di Wang, Shang-shang Jiang, Long Tang, Yue-fen Wang, Yuan Meng, Zhen Cai, Xue-yan Sun, Fang-qiang Cui, Wen-jing Zhao

**Affiliations:** a Department of Nephrology, Beijing Hospital of Traditional Chinese Medicine, Capital Medical University, Beijing, China; b Dongzhimen Hospital Affiliated to Beijing University of Chinese Medicine, Beijing, China

**Keywords:** Immunoglobulin A nephropathy, serum hemoglobin level, prognosis

## Abstract

**Background:**

Decreased serum hemoglobin (Hb) level is associated with Immunoglobulin A nephropathy (IgAN) progression. However, whether serum Hb level is an independent prognostic factor of IgAN remains controversial. Herein, we aimed to investigate the prognostic value of serum Hb level in IgAN.

**Methods:**

The Cochrane Library, Embase, PubMed and Open Grey databases were systematically searched and reviewed. Kidney disease progression of IgAN was defined as a doubling of serum creatinine (SCr), a 30% reduction in estimated glomerular filtration rate (eGFR), end-stage renal disease (ESRD), or death. We evaluated the hazard ratio (HR) between serum Hb level and the incidence of kidney disease progression in IgAN before and after adjusting for relevant covariates.

**Results:**

We included nine studies with 10006 patients in the meta-analysis. As a continuous variable, we found that serum Hb was an independent prognostic factor of IgAN [unadjusted HR = 0.89, 95% confidence interval (CI) = 0.84–0.95, *I*^2^ = 98%; adjusted HR = 0.85, 95% CI = 0.79–0.91, *I*^2^ = 0%]. The sensitivity analysis confirmed the stability of these results. Consistently, as a dichotomous variable defined as the below/above cutoff for anemia, we observed a positive correlation between serum Hb and kidney disease progression in IgAN (unadjusted HR = 2.12, 95% CI = 1.44–3.12, *I*^2^ = 79%; adjusted HR = 1.65, 95% CI = 1.20–2.27, *I*^2^ = 0%).

**Conclusion:**

Serum Hb level was independently correlated with the incidence of kidney disease progression in IgAN.

## 1. Introduction

Immunoglobulin A nephropathy (IgAN) is one of the most common primary glomerular diseases and a major etiology of end-stage renal disease (ESRD) worldwide [1,2]. After diagnosis, almost 40% of IgAN patients will develop ESRD within 20 years [3–5]. Therefore, early identification of prognostic factors and intervention of IgAN is critical and could effectively reduce ESRD risk.

Previous evidence has demonstrated that at the time of biopsy, the MEST score of the Oxford classification [refers to mesangial hypercellularity (M), endocapillary hypercellularity (E), segmental glomerulosclerosis (S), interstitial fibrosis/tubular atrophy (T) and the presence of crescents], proteinuria >1.0 g/d, hypertension, eGFR< 60 mL/min/1.73 m^2^, hypoproteinemia, hyperuricemia, high ratio of neutrophil/lymphocyte and lower bilirubin are independent risk factors of IgAN progression [5–10]. Anemia is common in renal failure patients. Low hemoglobin (Hb) levels and anemia are risk factors for CKD progression to ESRD and ESRD incidence in people without CKD [11]. Increasing studies have explored anemia in IgAN. While interstitial fibrosis and tubular atrophy are the strongest histological predictors of IgAN progression [12], a large cross-sectional study conducted in China found that anemia was associated with T2 in IgAN (tubulointerstitial renal lesions >50%), and the anemia degree was more severe than T0 (tubulointerstitial renal lesions ≤25%) and T1 (tubulointerstitial renal lesions: 26–50%) [13]. Moreover, some studies have suggested that decreasing Hb is independently associated with IgAN progression [14–16], while a few others did not confirm this finding [17,18]. Thus, the association between serum Hb level or anemia and IgAN prognosis remains controversial.

Herein, we performed a systematic review and meta-analysis to explore the value of serum Hb for the prognosis of IgAN patients.

## 2. Methods

### 2.1. Search strategy

Based on the terms and combinations, including ‘IGA Type Nephritis’, ‘Berger* disease’, ‘Immunoglobulin A Nephropathy’, ‘IGA nephropathy’, ‘Glomerulonephritis, IGA’, ‘HB’, ‘haemoglobin*’, ‘hemoglobin*’, ‘anaemia’ and ‘anaemia’, we systematically searched Cochrane Library, Embase, PubMed, and Open Grey databases from their inception to December 2022. We only included fully published studies in English and scanned the corresponding references for relevant studies. The protocol of this study was registered in PROSPERO (CRD 42021241208).

### 2.2. Inclusion and exclusion criteria

Studies were included as follows: for the participants (P): all participants should be more than 18 years old, and the IgAN should be confirmed by biopsy; for exposure (E): as a continuous variable: a decrease of 1 g/dL in serum Hb level; and as a dichotomous variable: below the cutoff for anemia); for the comparisons (C): as a dichotomous variable: above the cutoff for anemia; for the outcomes (O): a doubling of SCr, a 30% reduction in eGFR, death or ESRD, ESRD was defined as eGFR < 15 mL/min/1.73 m^2^ or the initiation of transplantation or dialysis; for the study design (S): it should be observational cohorts. Finally, the studies included in this analysis should have sufficient information for evaluating the risk ratio (RR) or HR and 95% confidence intervals (CIs). In contrast, we excluded non-cohort studies, conference abstracts, and studies without sufficient data. We performed this meta-analysis following the MOOSE checklist (Supplementary file 1).

### 2.3. Data collection and qualitative assessment

The information required for this analysis, such as kidney failure outcomes, the follow-up data, the usages of medication, proteinuria, serum Hb level, eGFR, country, age, sex, number of patients, year of publication, and the name of the first author, were independently extracted by two researchers (ZK and WMD). We used the Newcastle Ottawa quality assessment scale (NOS) to evaluate the quality of studies included in our analysis [19]. A NOS score >6 was considered a ‘high-quality’ study.

### 2.4. Statistical analysis

We conducted this study following The Cochrane Library handbook and the Quality of Reporting of Meta-Analysis guidelines [20,21]. Data analysis and graph formation were performed using the Review Manager 5.3 and the STATA 15.1 software. We extracted the risk estimates HR from each study using the Cox proportional hazards regressions. Unadjusted RR was calculated when the HRs were not reported. The fully adjusted and unadjusted risk coefficients for the association between serum Hb and kidney disease progression in IgAN were pooled and calculated. Serum Hb was analyzed as both a continuous and dichotomous variable (defined as the below/above cutoff for anemia). Moreover, in subgroup analyses for different gender, we pooled and calculated the fully adjusted risk coefficients for the association between serum Hb (as a dichotomous variable) and kidney disease progression in IgAN. Random-effect models were used to pool HRs. The heterogeneity of the studies was assessed using the chi-squared and *I*^2^ tests [22]. We assessed the influence of a single study on the overall risk estimate after one study was removed at a time to analyze the sensitivity. Publication bias was evaluated by Funnel plots, Begg’s and Egger’s tests as well as the trim-and-fill computation.

## 3. Results

### 3.1. Characteristics of selected studies

**Figure 1.**
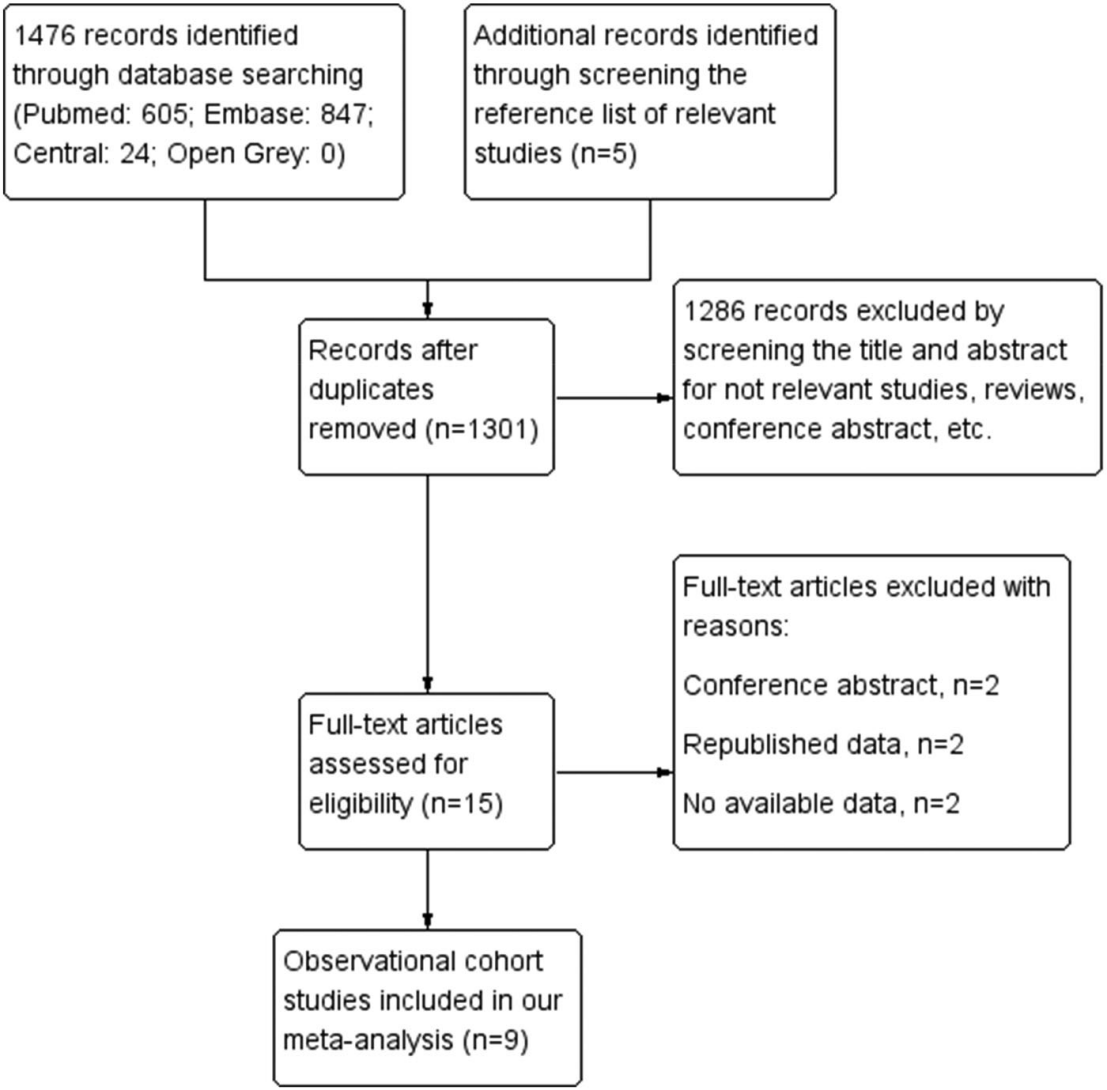
Flow diagram of study screening.

Initially, we obtained 1481 articles using our search strategy. Then, we excluded duplicated articles by titles and abstracts screening, and 1301 articles were considered potentially relevant. Among the 1301 articles, 1286 were excluded due to irrelevant or non-cohort studies. Through full-text reviews, two conference abstracts, two duplicated data-contained articles, and two articles without enough data to estimate the HR were removed from the remaining 15 articles. Finally, we included nine studies with 10,006 patients [10,14–18,23–25] (Figure 1).

In Table 1, among the nine studies, eight were performed in Asian countries: China, Korea, and Japan [10,14–16,18,23–25], and one was performed in Western countries (Turkey) [17]. Six of the nine studies estimated the correlation between the decreasing Hb in serum and kidney disease progression in IgAN [14–18,23], in which the HR was calculated using continuous variables relative to 1 g/dL. As a dichotomous variable (defined as the below/above cutoff for anemia), the correlation between serum Hb and kidney disease progression in IgAN was investigated by six studies [10,15,18,23–25]. All included studies were considered high quality because of their NOS score >6 (Supplementary file 2). The main limitation of these studies was the vague reporting of dropout rates and incomplete follow-ups. For males and females, Hb less than 12 and 11 g/dL were applied as a cutoff to define anemia in most studies, respectively.

**Table 1.** Characteristics of included studies.

Author/year	Country	Study type	Follow-up^a^ (yrs)	Selective criteria for anemia (g/dL)	Patients (*n*)	Age^a^ (yrs)	Men *n* (%)	eGFR^a^ (mL/min/1.73 m^2^)	Proteinuria^a^ (g/day)	Hemoglobin (g/dL)
Oh et al. [15] 2021	Korea	RCS	6.08	<13^b^ <12^c^	4326	39.3	2141 (49.5)	75	N.R.	13
Zhai et al. [16] 2021	China	PCS	1.17	N.R.	394	35	212 (54)	N.R.	1.86	12.8
Jiang et al. [10] 2021	China	RCS	5.1	<12^b^ <11^c^	1132	34.5	462 (40.81)	N.R.	2.0	N.R.
Yang et al. [18] 2020	China	RCS	3.6	<12^b^ <11^c^	642	33.23	281 (43.77)	85.84	1.43	13.3
Zhu et al. [24] 2020	China	RCS	7	<12^b^ <11^c^	1580	33.89	670 (42.41)	100.07	0.68	12.3
Lu et al. [23] 2020	China	RCS	2.08	<12^b^ <11^c^	193	33.9	74 (38.34)	90.6	1.32	12.98
Xie et al. [14] 2018	China	RCS	4.69	N.R.	934	36.5	462 (49.5)	74.7	0.12	12.9
Caliskan et al. [17] 2016	Turkey	PCS	2.8	N.R.	111	35	69 (62.2)	56	1.8	12.5
Tanaka et al. [25] 2015	Japan	RCS	4.9	<13^b^ <12^c^	694	36	330 (47.55)	80	1.24	13.5

Author/year	Events		UHR (URR) (95% Cl)	AHR (95% Cl)	Medication use, n (%)	Adjustments
	Anemia	Control				
Oh et al. [15] 2021	N.R.	N.R.	2.78 [2.353, 3.285]^&^ 0.768 [0.736, 0.802]^#^	1.675 [1.112, 2.522]^&^ 1.241 [0.871, 1.768]^b&^ 1.674 [1.097, 2.554]^c&^ 0.871 [0.773, 0.983]^#^	N.R.	Age, sex, diabetes mellitus, serum albumin, systolic blood pressure, smoking history, uric acid and time stratified eGFR, log(urine protein creatinine ratio), total cholesterol, C- reactive protein.
Zhai et al. [16] 2021	N.R.	N.R.	0.96 [0.95, 0.98]^#^	N.R.	N.R.	N.R.
Jiang et al. [10] 2021	N.R.	N.R.	N.R.	1.610 [0.962, 2.695]^&^	RASI 1132 (100); Immunosuppression therapy if necessary.	Age, sex, total bilirubin,hypertension, ALT, AST, uric acid, triglycerides, total cholesterol,number of erythrocytes in urine at high magnification, lactate dehydrogenase,nephrotic syndrome,Glucocorticoids,CKD stages,Oxford classification (MEST) score, and crescents.
Yang et al. [18] 2020	17/79	64/563	0.99 [0.98, 1.00]^#^ 1.89 [1.17, 3.06]^&^	N.R.	RASI 433(67.45); Immunosuppression therapy 378(58.88).	N.R.
Zhu et al. [24] 2020	80/389	103/1191	2.378 [1.818, 3.111]^&^	1.64 [1.01, 2.68]^b&^ 1.68 [1.02, 2.76]^c&^	RASI 1234 (78.1); Corticosteroids 1013 (64.36).	Age, 24-h proteinuria, eGFR, uric acid, mean arterial pressure, Oxford classification (MEST) score, crescents and treatments.
Lu et al. [23] 2020	4/36	6/157	0.968 [0.937, 1]^#^ 3.005 [0.846, 10.668]^&^	N.R.	N.R.	N.R.
Xie et al. [14] 2018	N.R.	N.R.	0.77 [0.72, 0.82]^#^	0.84 [0.77, 0.91]^#^	RASI 591 (73); Corticosteroids 448 (55.1).	N.R.
Caliskan et al. [17] 2016	N.R.	N.R.	N.R.	0.782 [0.559, 0.973]^#^	RASI 59 (53.2); Only prednisolone 25(22.5); Prednisolone + immunosuppressant 14 (12.6); Only Immunosuppressant 13 (11.7).	Age, sex, MAP, eGFR, Hgb, SCr, albumin, proteinuria, hematuria, Oxford classification, intensity and pattern of staining for C3, C1Q, IgA, IgG, IgM.
Tanaka et al. [25] 2015	32/334	43/508	1.132 [0.732, 1.751]^&^	N.R.	RASI 341 (49.1); Steriod 235 (33.9).	N.R.

UHR: unadjusted HR; AHR: adjusted HR; ^&^HR: calculated by comparing anemia with non-anemia IgAN patients for the incidence of kidney disease progression; ^#^HR: calculated using serum Hb level as a continuous variable for the incidence of kidney disease progression in IgAN patients; N.R.: not reported; PCS: prospective cohort study; RCS: retrospective cohort study; ^a^Median or mean; ^b^male; ^c^female; RASI: reninangiotensin system inhibitors.

### 3.2. Relationship of Hb as a continuous variable with IgAN outcomes

**Figure 2.**
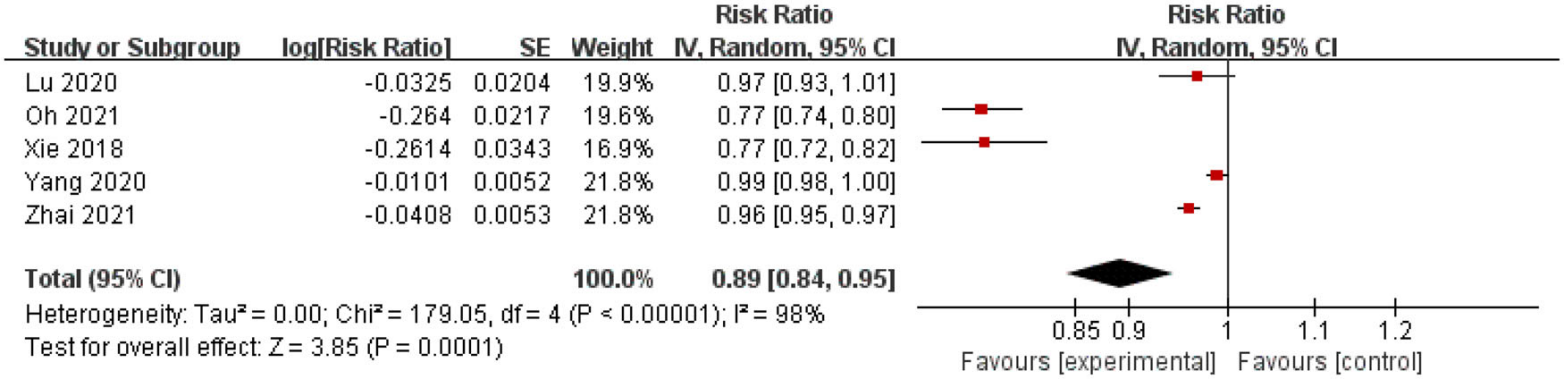
Forest plot of correlation between serum Hb and kidney disease progression in IgAN (unadjusted HR).

Five studies estimated the unadjusted HR of decreasing Hb by 1 g/dL with regards to kidney disease progression in IgAN [14–16,18,23]. A significant correlation between Hb and kidney disease progression in IgAN (HR = 0.89, 95% CI = 0.84–0.95) was observed using a random-effect model (Figure 2) with evidence of between-study heterogeneity (χ^2^ = 179.05, *I*^2^ = 98%).

**Figure 3.**
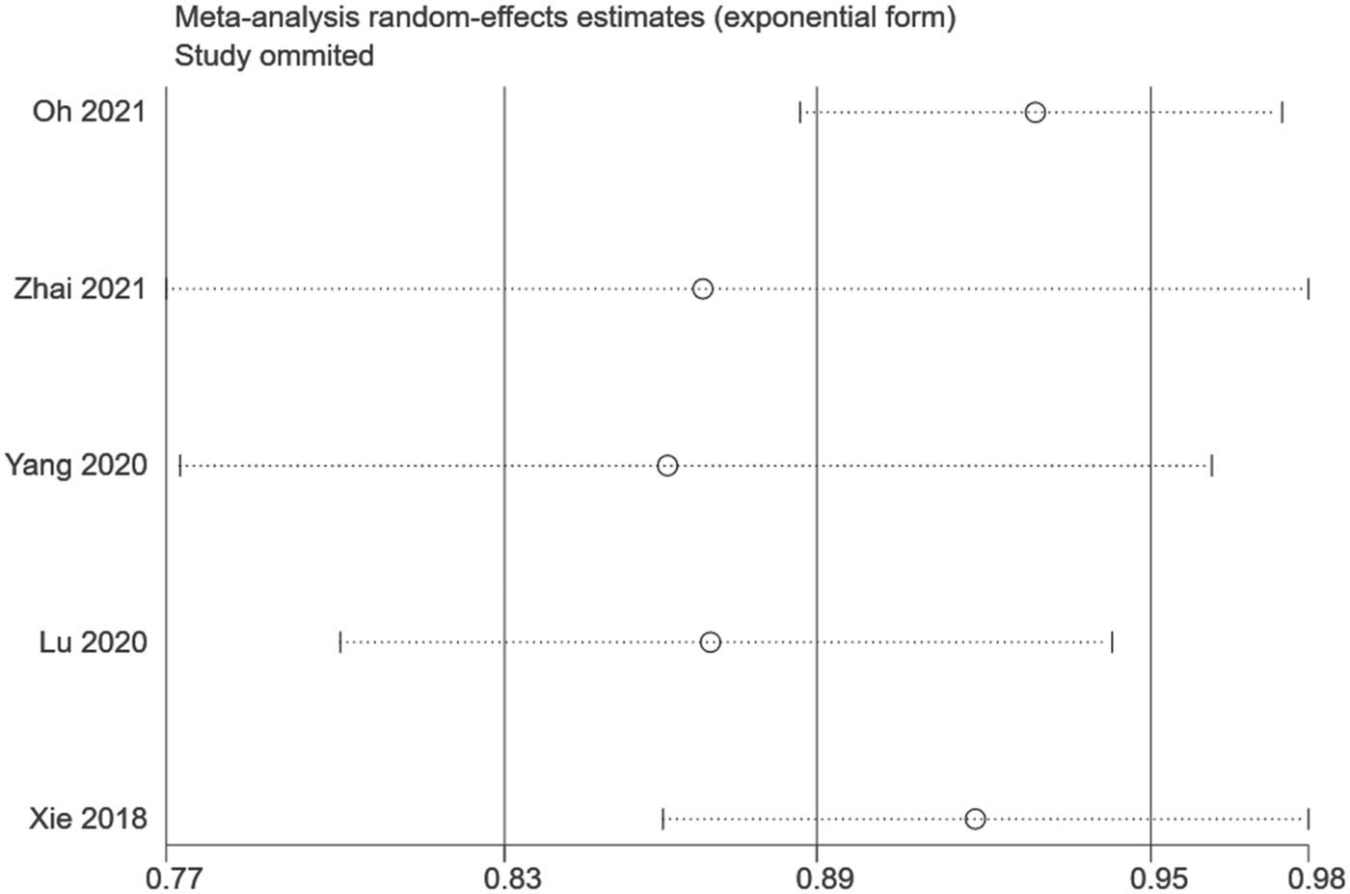
Sensitivity analyses.

To clarify the possible heterogeneity sources, we conducted a sensitivity analysis by removing one study at a time, and the correlation between serum Hb and kidney disease progression in IgAN was also confirmed (Figure 3).

**Figure 4.**
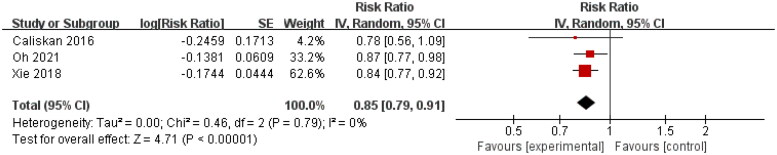
Forest plot of correlation between serum Hb and kidney disease progression in IgAN (adjusted HR).

Furthermore, three studies [14,15,17] calculated the adjusted HR to evaluate the relationship between serum Hb and kidney disease progression in IgAN. At least three potential confounders were adjusted in their studies. In the adjusted model, a significant correlation between serum Hb and kidney disease progression in IgAN was observed (HR = 0.85, 95% CI = 0.79–0.91) without evidence of heterogeneity (χ^2^ = 0.46, *I*^2^ = 0%) (Figure 4).

### 3.3. Relationship of Hb as a dichotomous variable with IgAN outcomes

**Figure 5.**
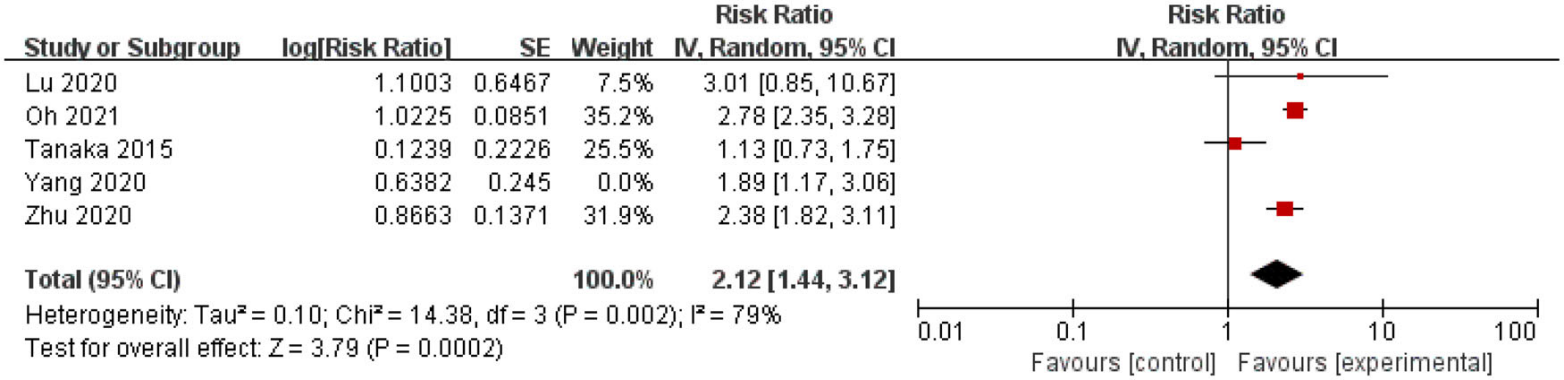
Forest plot of correlation between serum Hb (as a dichotomous variable) and kidney disease progression in IgAN (unadjusted HR).

Five studies calculated the unadjusted HR between serum Hb (as a dichotomous variable) and kidney disease progression in IgAN [15,18,23–25]. Serum Hb was also positively correlated with kidney disease progression in IgAN (HR = 2.12, 95% CI = 1.44–3.12, *I*^2^ = 79%) (Figure 5).

**Figure 6.**
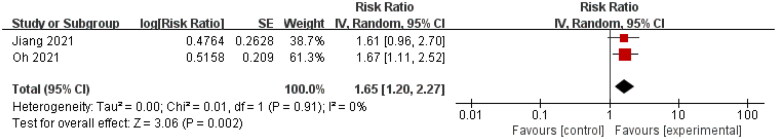
Forest plot of correlation between serum Hb (as a dichotomous variable) and kidney disease progression in IgAN (adjusted HR).

Two studies calculated the adjusted HR between serum Hb (as a dichotomous variable) and kidney disease progression in IgAN [10,15]. We also found a significant correlation between serum Hb and kidney disease progression in IgAN (HR = 1.65, 95% CI = 1.20–2.27) without evidence of heterogeneity (χ^2^ = 0.01, *I*^2^ = 0%) (Figure 6).

### 3.4. Consistency of the correlation between Hb and kidney disease progression in IgAN for different gender

**Figure 7.**
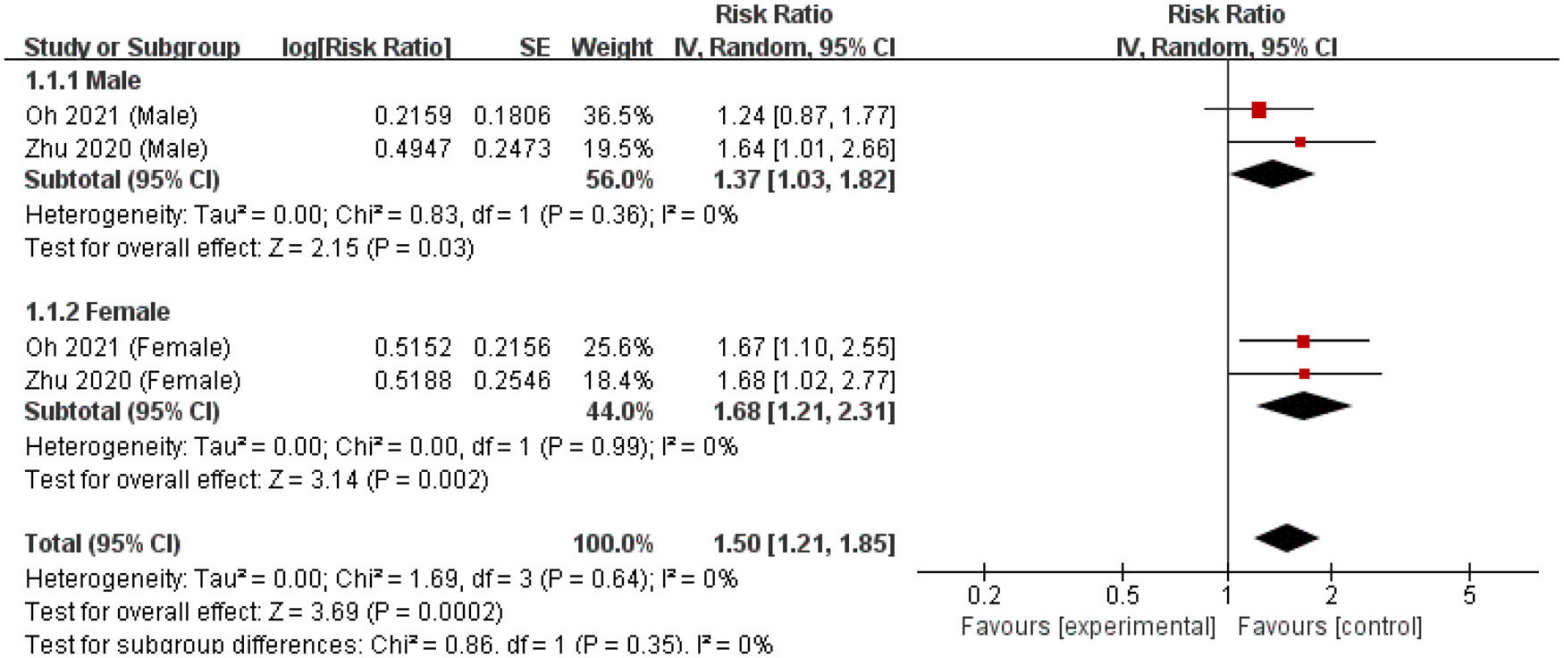
Forest plot of correlation between serum Hb and kidney disease progression in IgAN for different gender (adjusted HR).

Regarding the correlation between serum Hb (as a dichotomous variable) and kidney disease progression in IgAN in different gender, two included studies calculated the adjusted HR [15,24]. We found a positive correlation between serum Hb and kidney disease progression in IgAN for females (HR = 1.68, 95% CI = 1.21–2.31, *I*^2^ = 0%) and males (HR = 1.37, 95% CI = 1.03–1.82, *I*^2^ = 0%). The difference in the correlation between serum Hb and kidney disease progression caused by IgAN in males and females was not significant (test for subgroup difference, *I*^2^ = 0%) (Figure 7).

### 3.5. Publication bias

Funnel plots suggested no publication bias for the associations between serum Hb (as a continuous variable) and kidney disease progression in IgAN in the adjusted model (Supplementary file 5), the *p* values of Begg’s (*p* = 1.0) or Egger’s (*p* = 0.686) tests also suggested no publication bias. No trimming was needed based on the trim-and-fill test.

## 4. Discussion

As a chronic kidney disease (CKD) complication, renal anemia is common among IgAN patients (28.5%) [13]. Herein, we found independent associations of the increased kidney disease progression with decreased serum Hb in IgAN patients. In the subgroup analysis, the relationship between serum Hb with kidney disease progression of IgAN was consistent for different gender.

For a long time, it has been believed that renal hemodynamic changes (hyperfiltration theory of Brenner et al.) influenced the initiation and progression of glomerular sclerosis [26]. However, increasing studies have found that, compared to glomerular injury, renal function decline is significantly affected by tubulointerstitial injury [27–30]. In 2000, the damage caused by chronic ischemia of tubulointerstitium was defined as the final common pathway in ESRD (the chronic hypoxia hypothesis) by Fine et al. [31]. In the kidneys, oxygen delivery to the tubulointerstitium relies on the peritubular capillary plexus. Since the shunt and diffusion of oxygen between venous and arterial vessels that run in close parallel contact, the oxygen tensions of renal tissue are in fact comparatively low. Thus, the kidneys are sensitive to changes in systemic oxygenation [32]. Anemia might accelerate renal function decline by inducing tubulointerstitial hypoxia [33].

Tubulointerstitial hypoxia can induce the epithelial-mesenchymal transition (EMT) and increase α-smooth muscle actin and collagen I production, promoting tubulointerstitium fibrogenesis [34]. Besides, hypoxia can induce endothelial activation and cause leukostasis, thus leading to the blood flow compromission in peritubular capillary and reduced oxygen delivery [35]. Also, prolonged hypoxia might lead to mitochondria functional deficits and apoptosis of renal tubular cells [36]. Increased tubulointerstitium fibrosis extends the distance between the capillaries and tubular cells, reducing the oxygen diffusion efficiency and aggravating interstitial hypoxia [33]. Altogether, these changes institute a vicious cycle of regional hypoxia, exacerbating the loss of nephrons and accelerating ESRD progression.

Herein, the HRs were calculated and pooled before and after the adjustment for relevant covariates to investigate the association between serum Hb and kidney disease progression in IgAN. As a continuous variable, serum Hb significantly affected kidney disease progression in IgAN in both unadjusted and fully adjusted models. For pooling the unadjusted HRs, the random-effect models were conducted because of the significant heterogeneity (*I*^2^ = 98). The sensitivity analysis verified the stability of the results. For pooling the adjusted HRs, we did not detect the between-study heterogeneity (*I*^2^ = 0%). Similarly, as a dichotomous variable defined as the below/above cutoff for anemia, decreased serum Hb was significantly correlated with kidney disease progression in IgAN in both unadjusted and fully adjusted models. Further subgroup analyses for different gender using adjusted HRs confirmed the prognostic value of decreased serum Hb in IgAN-related kidney disease progression.

Although decreased Hb was significantly associated with poor prognosis of IgAN patients, whether early clinical intervention to renal anemia has renoprotective effects remains unknown. In IgAN, renal anemia is mainly caused by decreased erythropoietin (EPO) production [13]. Since 1989, erythropoiesis-stimulating agents (ESAs) have been applied to treat renal anemia [37,38]. ESAs have good curative effects in increasing Hb levels and reducing transfusion requirements. However, a large randomized controlled trial (RCT) conducted in 24 countries showed that in CKD patients who do not rely on dialysis, the use of darbepoetin alfa did not reduce ESRD risk and was associated with an increased risk of stroke [39]. As an inhibitor that specifically suppresses the enzymic activity of hypoxia-inducible factor prolyl hydroxylase enzyme, Roxadustat (FG-4592) has been demonstrated to be non-inferior to ESAs for improving hemoglobin in CKD patients [40,41]. Whether using FG-4592 will influence hard renal endpoints, including ESRD and mortality, remains unknown. Although an experimental study found that the HIF pathway might enhance kidney fibrosis [42], FG-4592 use did not suggest an increased risk of kidney fibrosis [43]. In contrast, in a mice model of ischemia/reperfusion（I/R）-induced acute kidney injury (AKI), FG-4592 protected tubular epithelial cells against hypoxia-induced injury by inhibiting inflammation [44]. Therefore, large RCTs are still needed to investigate whether HIF-PHI therapy at an earlier stage of Hb decline could delay the progression of kidney disease.

However, our current study also has some limitations. First, although we systematically reviewed databases, including grey literature, for the correlation between serum Hb (as a continuous variable) and kidney disease progression in IgAN, only three studies reported adjusted data. Besides, only two studies reported adjusted data between serum Hb (as a dichotomous variable) and kidney disease progression in IgAN. The lack of original studies might decrease the evidence strength of our findings. Second, although Begg and Egger’s tests did not indicate publication bias for the association between the decreasing Hb and IgAN-related kidney disease progression, potential bias risk was unavoidable. The limitation of the geographical region (most studies were conducted in Asia) and selective outcome reporting (lack of reports with negative results) might contribute to our findings’ potential bias risk. Third, we observed high heterogeneity for the association of decreasing Hb with kidney disease progression in IgAN patients in the unadjusted model (*I*^2^ = 98%). However, the stability of our results was verified through sensitivity analyses by excluding one study at a time. Moreover, the fully adjusted models suggested that serum Hb was an independent factor for IgAN prognosis as a continuous and dichotomous variable with no evidence of between-study heterogeneity (*I*^2^ = 0%).

## Conclusion

Our findings suggested an association between decreased serum Hb and kidney disease progression in IgAN patients. These observations demonstrated that decreased serum Hb might independently predict the prognosis of IgAN patients. Therefore, to clarify whether early clinical intervention for anemia might delay IgAN progression, high-quality RCTs are needed in the future.

## Supplementary Material

Supplemental Material
