# Supplementary material for: Is serum hemoglobin level an independent prognostic factor for IgA nephropathy?: a systematic review and meta-analysis of observational cohort studies
Source: Ren Fail. 2023 Jan 30;45(1):2171885. doi: 10.1080/0886022X.2023.2171885 (PMC9888460; doi:10.1080/0886022X.2023.2171885)
Supplement: Supplemental Material [file IRNF_A_2171885_SM9743.zip › IRNF 2171885/Supplementary file 2 NOS.pdf]

The NOS quality assessment of the included cohort studies

Study	Representativeness of the exposed cohort	Selection of the non exposed cohort	Ascertainment of exposure	Outcome of interest not present at start of study	Comparability of cohorts	Assessment of outcome	Follow-up long enough	Adequacy of follow up	Score
**Oh et al. [15] 2021**	YES	YES	YES	YES	YES	YES	YES	YES	8
**Zhai et al. [16] 2021**	YES	YES	YES	YES	NO	YES	YES	NO	6
**Jiang et al. [10] 2021**	YES	YES	YES	YES	YES	YES	YES	NO	7
**Yang et al. [18] 2020**	YES	YES	YES	YES	NO	YES	YES	YES	7
**Zhu et al. [24] 2020**	YES	YES	YES	YES	YES	YES	YES	YES	8
**Lu et al. [23] 2020**	YES	YES	YES	YES	NO	YES	YES	NO	6
**Xie et al. [14] 2018**	YES	YES	YES	YES	NO	YES	YES	NO	6
**Caliskan et al. [17] 2016**	YES	YES	YES	YES	YES	YES	YES	NO	7
**Tanaka et al. [25] 2015**	YES	YES	YES	YES	YES	YES	YES	YES	8

YES: the study meet the item in the scale; NO: the study didn’t mentioned it (unclear) or failed to meet that item.
