# Supplementary material for: Is serum hemoglobin level an independent prognostic factor for IgA nephropathy?: a systematic review and meta-analysis of observational cohort studies
Source: Ren Fail. 2023 Jan 30;45(1):2171885. doi: 10.1080/0886022X.2023.2171885 (PMC9888460; doi:10.1080/0886022X.2023.2171885)
Supplement: Supplemental Material [file IRNF_A_2171885_SM9743.zip › IRNF 2171885/Supplementary file 3.pdf]

Definition and measurement method of the outcomes of interest.

**Author/year**	Outcomes	Definition of outcomes	Formula to calculate eGFR	Method to measure Scr
**Oh et al. [15] 2021**	IgAN progression.	IgAN progression: 1) 2-fold increase in baseline serum creatinine; or 2) 50% decline in the eGFR; 3) the initiation of dialysis; or 4) kidney transplantation.	MDRD	N.R.
**Zhai et al. [16] 2021**	1) doubling of Scr; or 2) ESRD; or 3) death; or 4) 30% reduction in eGFR.	ESRD: 1) eGFR<15 ml/min/1.73 m^2; or 2) initiation of renal replacement therapy, including haemodialysis, peritoneal dialysis or renal transplantation.	N.R.	The sarcosine oxidase enzymatic method.
**Jiang et al. [10] 2021**	1) a 50% decline in e-GFR; or 2) ESKD; or 3) renal transplantation; or 4) death.	ESKD: 1) eGFR≤15 ml/min/1.73 m^2; or 2) maintenance renal replacement treatment.	CKD-EPI	N.R.
**Yang et al. [18] 2020**	1) eGFR decline ≥50%; or 2) doubling of Scr; or 3) ESRD.	ESRD: 1) eGFR<15 ml/min/1.73 m^2; or 2) initiation of dialysis or transplantation.	CKD-EPI	N.R.
**Zhu et al. [24] 2020**	1) all cause death; or 2) kidney failure.	Kidney failure: 1) eGFR decline＞40%; or 2) ESKD (eGFR<15 ml/min/1.73 m^2 or need for renal replacement therapy, including haemodialysis, peritoneal dialysis, or renal transplantation).	CKD-EPI	N.R.
**Lu et al. [23] 2020**	1) initiation of dialysis; or 2) doubling of Scr.	N.R.	CKD-EPI	N.R.
**Xie et al. [14] 2018**	ESRD	ESRD: initiation of dialysis or transplantation.	CKD-EPI	N.R.
**Caliskan et al. [17] 2016**	1) Kidney failure; or 2) eGFR decline ≥50%.	Kidney failure: eGFR <15 ml/min per 1.73 m^2.	CKD-EPI	The enzymatic method.

**Tanaka et al. [25] 2015**	ESRD	ESRD: the initiation of renal replacement therapy, including hemodialysis, peritoneal dialysis and kidney transplantation.	IDMS-MDRD	The Jaffe method or the enzymatic method. The Jaffe method were converted to values for the enzymatic method by subtracting 0.207 mg dl^{-1}.

CKD-EPI: Chronic Kidney Disease Epidemiology Collaboration (CKD-EPI) equation; MDRD: Modification of Diet in Renal Disease equation; IDMS-MDRD: the modified isotope dilution mass spectorometry - modification of diet in renal disease for Japanese; N.R. : not reported.
