# Supplementary material for: Is serum hemoglobin level an independent prognostic factor for IgA nephropathy?: a systematic review and meta-analysis of observational cohort studies
Source: Ren Fail. 2023 Jan 30;45(1):2171885. doi: 10.1080/0886022X.2023.2171885 (PMC9888460; doi:10.1080/0886022X.2023.2171885)
Supplement: Supplemental Material [file IRNF_A_2171885_SM9743.zip › IRNF 2171885/Supplementary file 4 Search strategy.pdf]

Search strategies.

Pubmed:
#1 Anemia/
#2 (anemia or anaemia or hemoglobin* or haemoglobin* or HB)
#3 (#1 or #2)
#4 Glomerulonephritis,IGA/
#5 (Glomerulonephriti* IGA or Berger* disease or IGA nephropathy or Immunoglobulin A Nephropathy or IGA Type Nephritis)
#6 (#4 or #5)
#7 (#3 and #6)

Embase:
#1 Anemia/
#2 (anemia or anaemia or hemoglobin* or haemoglobin* or HB).mp. [mp=title, abstract, heading word, drug trade name, original title, device manufacturer, drug manufacturer, device trade name, keyword, floating subheading word, candidate term word]
#3 (#1 or #2)
#4 Glomerulonephritis, IGA/
#5 (Glomerulonephriti* IGA or Berger* disease or IGA nephropathy or Immunoglobulin A Nephropathy or IGA Type Nephritis).mp. [mp=title, abstract, heading word, drug trade name, original title, device manufacturer, drug manufacturer, device trade name, keyword, floating subheading word, candidate term word]
#6 (#4 or #5)
#7 (#3 and #6)

Central:
#1 Anemia/
#2 (anemia or anaemia or hemoglobin* or haemoglobin* or HB).mp. [mp=title, original title, abstract, mesh headings, heading words, keyword]
#3 (#1 or #2)
#4 Glomerulonephritis, IGA/
#5 (Glomerulonephriti* IGA or Berger* disease or IGA nephropathy or Immunoglobulin A Nephropathy or IGA Type Nephritis).mp. [mp=title, original title, abstract, mesh headings, heading words, keyword]
#6 (#4 or #5)
#7 (#3 and #6)

Open Grey:
(Glomerulonephritis, IGA OR Glomerulonephriti* IGA OR Berger* disease OR IGA nephropathy OR Immunoglobulin A Nephropathy OR IGA Type Nephritis) AND (Anemia OR anemia or anaemia or hemoglobin* or haemoglobin* or HB)
