# Supplementary material for: Is serum hemoglobin level an independent prognostic factor for IgA nephropathy?: a systematic review and meta-analysis of observational cohort studies
Source: Ren Fail. 2023 Jan 30;45(1):2171885. doi: 10.1080/0886022X.2023.2171885 (PMC9888460; doi:10.1080/0886022X.2023.2171885)
Supplement: Supplemental Material [file IRNF_A_2171885_SM9743.zip › IRNF 2171885/Supplementary_file_1_MOOSE new.pdf]

The MOOSE Checklist of the article

Criteria		Brief description of how the criteria were handled in the meta-analysis
**Reporting of background should include**		
√	Problem definition	Decreased serum hemoglobin (Hb) level has been reported to be associated with the progression of IgA nephropathy (IgAN). However, whether serum Hb is an independent prognostic factor of IgAN remains controversial.
√	Hypothesis statement	Serum Hb level may be an independent prognostic factor for IgA nephropathy.
√	A statement of objectives that includes the study population, the condition of interest, the exposure or intervention, and the outcome(s) considered	Population: adult patients (> 18 years old) with biopsy-proven IgA nephropathy.<br>Condition of interest: observational cohort studies.<br>Exposures: as a continuous variable: a decrease of 1 g/dL in serum Hb level; as a dichotomous variable: below the cut-off for anemia.<br>Outcome: doubling of the serum creatinine level, 30% reduction in eGFR, ESRD, or death.
**Reporting of search strategy should include**		
√	Qualifications of searchers	The credentials of two investigators ZK and WMD are indicated in the author list.
√	Search strategy, including time period included in the synthesis and keywords	Supplementary file 4 shows the search strategy of the study. Databases were systematically reviewed from inception to December 2022. Keywords list in the method part of the article.
√	Databases and registries searched	PubMed, Embase, Cochrane Library, and Open Grey were searched.
√	Search software used, name and version, including special features	We did not employ a search software. EndNote was used to merge retrieved citations and eliminate duplications.
√	Use of hand searching	We hand-searched bibliographies of retrieved papers for additional references.
√	List of citations located and those excluded, including justifications	Details of the literature search process are outlined in the flow chart. The citation list is available upon request.
√	Method of addressing articles published in languages other than English	Only studies published in English were included in our meta-analysis.
√	Method of handling abstracts and unpublished studies	Only published studies were included in our analysis.
√	Description of any contact with authors	We have tried our best to contact with authors for some additional data, only Professor Fu, the corresponding author of “Yang 2020”

		provided some data to us, we have put it in table 1+.
	**Reporting of methods should include**	
√	Description of relevance or appropriateness of studies assembled for assessing the hypothesis to be tested	Detailed inclusion and exclusion criteria were described in the methods section.
√	Rationale for the selection and coding of data	Data extracted from each of the studies were relevant to the population characteristics, study design, exposure, outcome, and possible confounding factors of the association. Two review authors (ZK and WMD) will independently extract data. Any discrepancies that arise will be resolved by discussion, or by consulting a third author. A standardized form will be used to extract data from the included studies. This data will be assessed for quality, and then analyzed for synthesis of conclusions. If data from the selected studies is deemed to be missing, the appropriate study author will be contacted for clarification.
√	Assessment of confounding	Hazard ratio (HR) between serum Hb level and kidney disease progression of IgA nephropathy were evaluated before and after adjustment for known covariates. Serum Hb was analyzed as both a continuous and dichotomous variable (defined as the below/above cut-off for anemia). The HR between serum Hb level (as a dichotomous variable) and the incidence of kidney disease progression in IgAN in different gender was evaluated after adjustment for relevant covariates. All adjusted confounding factors were listed in Table 1. In the adjusted model, at least three potential confounders were adjusted for pooled-analysis.
√	Assessment of study quality, including blinding of quality assessors; stratification or regression on possible predictors of study results	Study quality was assessed by using New-Ottawa scale.<br>Subgroup analyses and sensitivity analysis were conducted.
√	Assessment of heterogeneity	Heterogeneity of the studies were explored within two types of study designs using Cochrane's Q test of heterogeneity and I^2 statistic that provides the relative amount of variance of the summary effect due to the between-study heterogeneity.
√	Description of statistical methods in sufficient detail to be replicated	Description of methods of meta-analyses, sensitivity analyses and assessment of publication bias are detailed in the methods.
√	Provision of appropriate tables and	We included 1 flow chart detailing the terms used for database

	graphics	search and study selection, 4 forest plots for association between serum Hb and kidney disease progression in IgAN, 1 forest plot for association between serum Hb and IgAN-caused kidney disease progression according to sex, 1 figure for sensitivity analysis, 1 funnel plot for publication bias, 1 table for the characteristics of the included studies, 1 table for the definition and method of measurement of the outcome of interest.
**Reporting of results should include**		
√	Graph summarizing individual study estimates and overall estimate	Figure 2, 4, 5, 6, 7.
√	Table giving descriptive information for each study included	Table 1.
√	Results of sensitivity testing	Detailed sensitive testing was described in the results section (Figure 3).
√	Indication of statistical uncertainty of findings	95% confidence intervals were presented with all summary estimates. Potential reasons for the observed heterogeneity were discussed.
**Reporting of discussion should include**		
√	Quantitative assessment of bias	The assessment of publication bias was performed both visually inspected funnel plot and statistically tested for funnel asymmetry using the Begg's and Egger's regression tests. Moreover, trim and fill methods were used to calibrate for publication bias.
√	Justification for exclusion	The exclusion criteria were described in the method section. The process of exclusion was listed on Figure 1.
√	Assessment of quality of included studies	Study quality was assessed by using New-Ottawa scale.
**Reporting of conclusions should include**		
√	Consideration of alternative explanations for observed results	For IgA nephropathy patients with impaired renal function, anemia is one of the most common complications, which could in turn further exacerbate kidney function. However, evidence from observational studies only shows an association between decreased serum Hb and kidney disease progression in IgAN, not a cause-and-effect relationship.
√	Generalization of the conclusions	Our meta-analysis indicates that IgAN patients with decreased serum Hb might increase the risk of kidney disease progression during follow-up.

√	Guidelines for future research	High-quality randomized controlled trials are required to determine whether early prevention and timely control of decreased serum Hb may delay kidney disease progression in patients with IgA nephropathy.
√	Disclosure of funding source	No separate funding was necessary for the undertaking of this systematic review.
