# Supplementary figures and images for: Is serum hemoglobin level an independent prognostic factor for IgA nephropathy?: a systematic review and meta-analysis of observational cohort studies
Source: Ren Fail. 2023 Jan 30;45(1):2171885. doi: 10.1080/0886022X.2023.2171885 (PMC9888460; doi:10.1080/0886022X.2023.2171885)

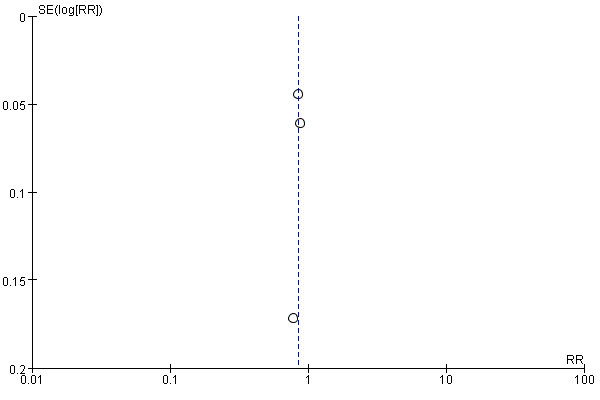

Supplement: Supplemental Material [file IRNF_A_2171885_SM9743.zip › IRNF 2171885/Supplementary file 5 Funnel plots for publication bias.png]
